# Destabilization of NaBH_4_ by Transition Metal Fluorides

**DOI:** 10.3390/molecules25040780

**Published:** 2020-02-12

**Authors:** Isabel Llamas Jansa, Georgios N. Kalantzopoulos, Kari Nordholm, Bjørn C. Hauback

**Affiliations:** Department for Neutron Characterization, Institute for Energy Technology, P.O. Box 40, NO-2027 Kjeller, Norway; georgios.kalantzopoulos@kjemi.uio.no (G.N.K.); kari.kj.no88@gmail.com (K.N.); Bjorn.Hauback@ife.no (B.C.H.)

**Keywords:** sodium borohydride, transition metal fluoride, hydrogen storage, destabilization, additives, future fuels, renewable energy

## Abstract

With the goal of improving performance of a hydrogen-rich storage medium, the influence of a collection of first and second period transition metal fluorides on the destabilization of NaBH_4_ is studied on samples produced by ball milling NaBH_4_ with 2 mol% of a metal fluoride additive. The effects obtained by increasing additive amount and changing oxidation state are also evaluated for NbF_5_, CeF_3_, and CeF_4_. The as-milled products are characterized by in-house power X-ray diffraction, while the hydrogen release and decomposition are monitored by temperature programmed desorption with residual gas analysis, differential scanning calorimetry, and thermogravimetry. The screening of samples containing 2 mol% of additive shows that distinctive groups of transition metal fluorides affect the ball milling process differently depending on their enthalpy of formation, melting point, or their ability to react at the temperatures achieved during ball milling. This leads to the formation of NaBF_4_ in the case of TiF_4_, MnF_3_, VF_4_, CdF_2_, NbF_5_, AgF, and CeF_3_ and the presence of the metal in CrF_3_, CuF_2_, and AgF. There is no linear correlation between the position of the transition metal in the periodic table and the observed behavior. The thermal behavior of the products after milling is given by the remaining NaBH_4_, fluoride, and the formation of intermediate metastable compounds. A noticeable decrease of the decomposition temperature is seen for the majority of the products, with the exceptions of the samples containing YF_3_, AgF, and CeF_3_. The largest decrease of the decomposition temperature is observed for NbF_5_. When comparing increasing amounts of the same additive, the largest decrease of the decomposition temperature is observed for 10 mol% of NbF_5_. Higher amounts of additive result in the loss of the NaBH_4_ thermal signal and ultimately the loss of the crystalline borohydride. When comparing additives with the same transition metal and different oxidation states, the most efficient additive is found to be the one with a higher oxidation state. Furthermore, among all the samples studied, higher oxidation state metal fluorides are found to be the most destabilizing agents for NaBH_4_. Overall, the present study shows that there is no single parameter affecting the destabilization of NaBH_4_ by transition metal fluorides. Instead, parameters such as the transition metal electronegativity and oxidation state or the enthalpy of formation of the fluoride and its melting point are competing to influence the destabilization. In particular, it is found that the combination of a high metal oxidation state and a low fluoride melting point will enhance destabilization. This is observed for MnF_3_, NbF_5_, NiF_2_, and CuF_2_, which lead to high gas releases from the decomposition of NaBH_4_ at the lowest decomposition temperatures.

## 1. Introduction

As hydrogen becomes one of the important alternative energy carriers for renewable energy sources, the discussion about its safe and efficient storage gains momentum. The challenge is not only to achieve small compact systems with high gravimetric and volumetric hydrogen densities fulfilling the necessary safety requirements, but for a competitive practical use, the hydrogen needs to be efficiently absorbed and desorbed. These goals can be reached by utilizing storage media that have intrinsically high hydrogen densities such as pressurized cylinders and cryogenic liquid hydrogen systems, as well as by solid-state hydrogen containing materials. The latter method has the additional advantages of safety and high volumetric density [1,2,3,4,5,6,7].

Among solid-storage materials, first and second group borohydrides (LiBH_4_, NaBH_4_, Ca(BH_4_)_2_, and Mg(BH_4_)_2_) [8,9,10,11,12] have been for two decades very attractive candidates because of their gravimetric densities of the order of 10 to 20 wt% H_2_ [13,14,15]. NaBH_4_, which has a high gravimetric capacity of 10.6 wt% and a decomposition temperature of about 535 ${}^{\circ }$C [16], has gone from being a favorite solid-storage material in the early 2000s to being rejected by the U.S. Department of Energy (DoE) [17] for on-board applications, to then again being described as a fuel for the future [18] due to its large yields of hydrogen release by hydrolysis and thermal decomposition that can be readily used in aqueous solutions in some types of fuel cells such as proton exchange membrane fuel cells (PEMFCs) or direct boron hydride fuel cells (DBFCs) [18,19,20,21].

The present work focuses on reducing the thermal desorption temperature of NaBH_4_ below 535 ${}^{\circ }$C by adding small amounts of transition metal fluorides (TMFs). This is an extension of two previous works: one with transition metal chlorides (TMCs) that showed the formation of NaBH_4_-chloride substituted phases [22] and another with selected TMFs, where no substitution was found [23].

An extensive literature review including the last four decades reveals a limited amount of work concerning the effect of fluorides on borohydrides. The first report of a borohydride being ball-milled with a variety of fluorides corresponds to Zhang et al. [24]. In this work, selected chlorides were found to form new borohydrides easier than their corresponding fluorides. Al-Kukhun et al. [25] and Zhang et al. [26] also found that the addition of selected fluorides (NbF_5_ and CaF_2_ and ZnF_2_ and TiF_3_, respectively) to MgBH_4_ had a positive effect on the hydrogen release and the kinetics of the borohydride. Furthermore, Minella et al. [27] investigated the sorption properties and reversibility of the Ti(IV) and Nb(V) doped-(CaBH_4_)_2_-MgH_2_ system. Adding NbF_5_ resulted in a system with enhanced reversibility by slightly suppressing the formation of CaB_12_H_12_. Likewise, Zhou et al. [28] used CeF_3_ as a catalyst on LiBH_4_ nanoconfined on activated carbon. They found a considerable decrease of the onset temperature of hydrogen release and a substantial increase in the dehydrogenation capacity. The fluoride substitution of LiBH_4_ by Richter et al. [29] was one of the most significant destabilization effects of fluorides on borohydrides observed up to date.

The first study involving NaBH_4_ and fluorides did not occur until early 2013 when Rude et al. [30] reported fluorine substitution on NaBH_4_ while investigating different NaBH_4_-NaBF_4_ mixtures. The fluorine-substituted phases were found to decompose into more stable compounds, while the NaBH_4_-NaBF_4_ composite itself presented considerably lower decomposition temperature. Chong et al. [31] found that the addition of LaF_3_ to NaBH_4_ promoted hydrogen sorption better than LaH.

The literature about the co-addition of more than one transition metal fluoride to a borohydride is rare. Recently, Huang et al. [32] found that adding ScF_3_ and YF to a NaBH_4_-containing system resulted in a three step hydrogen desorbing system with enhanced reversibility when using the two fluorides simultaneously. The results were partially confirmed by Zhao et al. [33] on the reversibility of 3NaBH_4_/ScF_3_ and by Huang et al. [34] on the reversible hydrogen sorption behavior of 3NaBH_4_-(x) YF_3_-(1 − x) GdF_3_. A recent review by Jain et al. [35] summarized the catalytic effect on lightweight hydrogen storage materials of a variety of compounds, including TiF_3_, TiF_4_, CeF_4_, NbF_5_, ZrF_4_, ternary K-TM-F fluorides (TM: Ti, Zr, Ni, Fe), NaMgF_3_, and NaF.

Additionally, Mao et al. [36] concluded that using metal fluorides as additives is a promising direction for improving the sorption kinetics of NaBH_4_ by lowering the energy barriers. These authors stated that both Ti and F have a positive effect. However, the physical, chemical, or thermodynamic parameters of the halide responsible for the increase in the decomposition rate of NaBH_4_/borohydrides/hydrides have so far not been identified. It is generally suggested that the oxidation state of the metal element that forms the halide plays the most important role. This is justified by the influence of different catalysts observed for chemical compounds that exist in only one oxidation state when comparing to chemical compounds with multivalent metals [37,38]. Similar discussions have been carried out about the influence of the oxidation state of the metal for catalyzed MgH_2_ [39,40].

All these studies showed that some metal fluorides induce strong effects on the destabilization of particular borohydrides. However, there is still a large number of fluorides whose effect on borohydrides has not been reported that might be beneficial for the hydrogen storage community. Continuing with this line of investigation, the main focus of the present work is to study the destabilization effects of available transition metal fluorides (TMFs) from the first and second periods of the periodic table on NaBH_4_. These effects might occur through the formation of new compounds, as well as the mechanochemical process itself.

The ball-milled products are analyzed by powder X-ray diffraction (PXD) and a variety of thermal methods including differential scanning calorimetry (DSC) and temperature-programmed desorption (TPD). The observed behavior is discussed in terms of the transition metal (TM) electronic structure and the position in the periodic table, as well as the ability of the fluoride to react during milling and form new compounds.

Variations due to the additive amount and oxidation state are also discussed. In particular, NbF_5_ was chosen as one of the additives based on previous results by Luo et al. [41] showing an increase of solubility during ball milling due to its low melting point (90 ${}^{\circ }$C). On the other hand, increasing the oxidation state of a metal has been shown to lead to compounds with a lower melting point and, therefore, higher solubility during the ball milling process [41]. This is tested by using CeF_3_ and CeF_4_ as additives to NaBH_4_.

## 2. Results and Discussion

### 2.1. Ball Milling Effects of the TMFs on NaBH_4_

**Figure A1:**
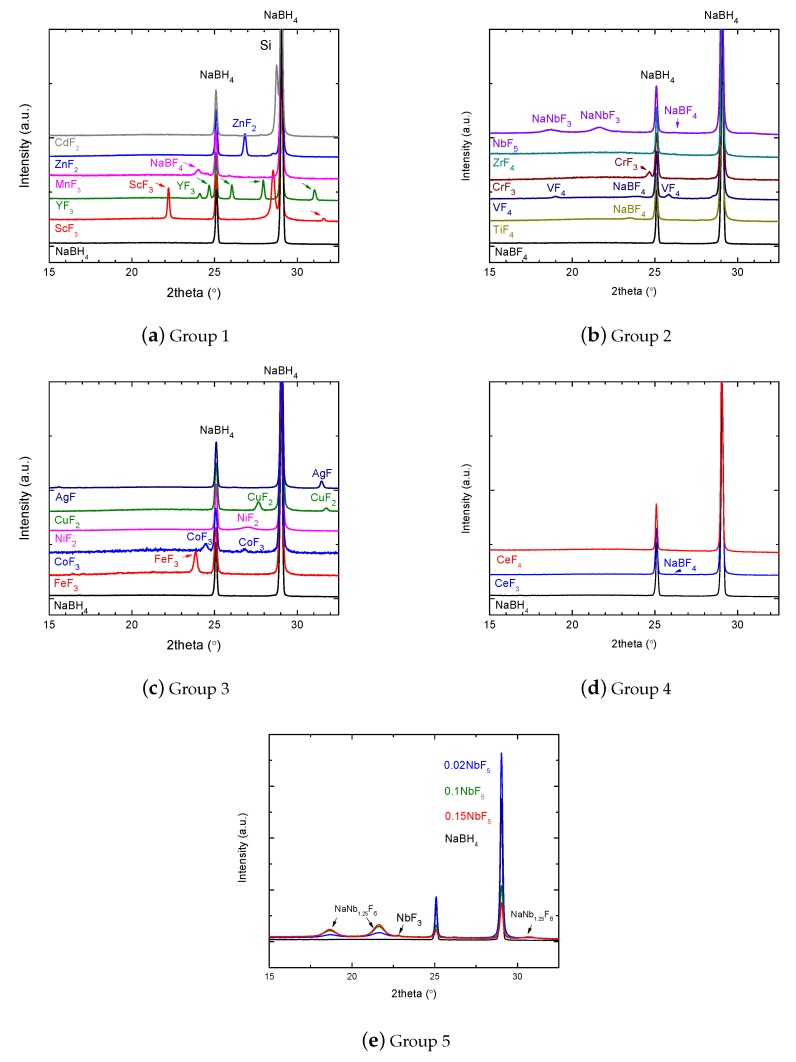
PXD data corresponding to the samples distributed in the different groups discussed in the text.

Table 1 summarizes the PXD data obtained for all the samples after ball milling and analyzed by DIFFRAC plus EVA in terms of the wt% content of the different compounds in the mixture. The table also contains the calculated wt% of the original mixtures for comparison. The same data are included in Appendix A as PXD plots (Figure A1). The data showed that all the samples still contained crystalline NaBH_4_ in large amounts after milling, although the exact composition of the products varied depending on the TM fluoride. Moreover, the lack of a shift in the Bragg peaks corresponding to the remaining NaBH_4_ indicated that there was no substitution in the NaBH_4_ unit cell despite the presence of crystalline NaBF_4_ in some of the samples. This was in agreement with the previously reported formation of NaBF_4_ [23].

The added fluorides remained as a crystalline phase for ScF_3_, FeF_3_, CrF_3_, NiF_3_, CoF_3_, CuF_2_, VF_4_, ZnF_2_, CdF_2_, YF_3_, and AgF. With the exception of AgF and VF_4_, these were all fluorides with melting points above 800 ${}^{\circ }$C. On the other hand, TiF_4_, MnF_3_, NbF_5_, ZrF_4_, CeF_4_, and CeF_3_ did not appear as crystalline phases in the PXD results, and no peaks corresponding to NbF_5_ were seen in the 10 and 15 mol% cases either. Except for the ZrF_4_ and CeF_4_ containing samples, which only showed crystalline NaBH_4_ in the PXD pattern, the disappearance of the fluoride in these samples was correlated with the appearance of NaBF_4_ and/or metallic TM. The presence of other compounds containing TM and fluorine could not be confirmed with the current PXD data.

A detailed analysis of the PXD patterns suggested a classification of the samples based on the products of the ball milling. First, ScF_3_, FeF_3_, NiF_2_, ZnF_2_, and YF_3_ showed no effect on the milling process. For these additives, the original ratio between NaBH_4_ and the fluoride was still present in the powder after ball milling. Small changes of the composition appeared for the samples containing CrF_3_, CoF_3_, and CuF_2_. This was seen by the presence of metallic Cr and Cu, respectively, while for CoF_3_, the presence of CoF_2_ was likely related to the original fluoride. Stronger changes of the composition were introduced by CdF_2_, CeF_3_, and AgF. In these cases, NaBF_4_ was present in the products together with the original NaBH_4_ and the fluoride. For the AgF case, metallic Ag and Ag_2_F were also seen in the PXD pattern.

TiF_4_, MnF_3_, VF_4_, and NbF_5_ (2, 10, and 15 mol%) produced the strongest changes of the composition of the samples after milling. This was mostly seen for TiF_4_, MnF_3_, and VF_4_ by a significant amount of NaBF_4_ and for VF_4_ by the additional metallic V. For the NbF_5_ cases, the amount of NaBF_4_ produced by milling was smaller than published earlier [23]. On the other hand, two new compounds containing Nb appeared in these samples: NbF_3_ and NaNb_1.25_F_6_. The content of these two products increased with NbF_5_ content in the mixture. The presence of F${}^{-}$ containing compounds in some of the studied cases confirmed the decomposition of the original fluorides and some level of H${}^{-}$ substitution in small amounts of NaBH_4_.

In contrast, CeF_4_ and ZrF_4_ containing samples showed only crystalline NaBH_4_ after milling, with the exception of a nonsymmetric peak at 25${}^{\circ }$ for CeF_4_ indicating a substituted phase. The comparison of the composition of the samples with CeF_3_ and CeF_4_ after milling showed that both oxidation states led to the disappearance of the fluoride in the crystalline form. Moreover, for CeF_3_, the analysis showed that the fluoride decomposed to form NaBF_4_, while for CeF_4_, there was no crystalline indication of the fluoride dissociating (it could still be there as amorphous). The effect of the oxidation state could also be seen by comparing the reported use of TiF_3_ [23] and the current use of TiF_4_. While the published data showed no formation of a new crystalline phase after ball milling for 3 h, the current data showed the formation of up to 18 wt% of NaBF_4_ after only 1 h ball milling, when using the higher oxidation state.

Overall, the melting point of the fluoride seemed to play a role in the interaction with NaBH_4_ during ball milling. This was easily seen in the case of NbF_5_, but also in the general trends observed between samples that contained high melting point TM fluorides such as ScF_3_ and NiF_2_, which led to mostly unchanged sample compositions, and those with lower melting points such as TiF_4_ and VF_4_, which led to the formation of NaBF_4_. However, the PXD results alone did not establish any correlation between the ability of the fluoride to interact chemically with NaBH_4_ and properties such as the enthalpy of formation, the electronic structure, or the oxidation state of the TM.

### 2.2. Effect of TMFs on the Destabilization of NaBH_4_

#### 2.2.1. Pure NaBH_4_ with Different Calorimetry Methods

**Figure 1:**
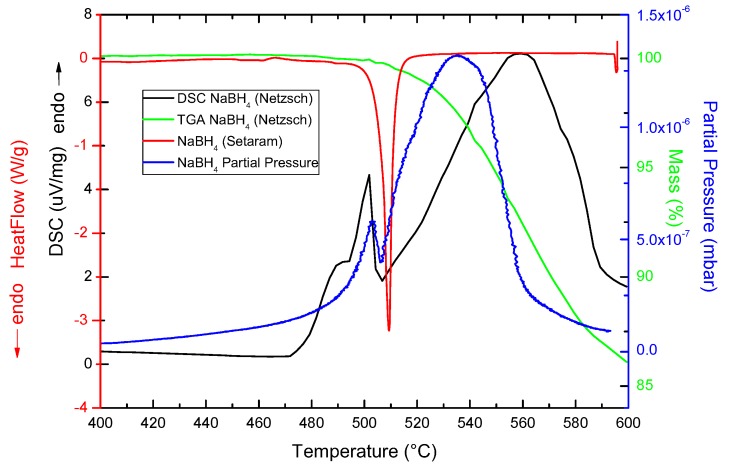
Comparison of the different calorimetry and gravimetric methods used to measure NaBH_4_. Black: DSC-Netzsch in μV/mg; red: DSC-Setaram in W/g; blue: TPD in mbar of H_2_; green: TGA in mass % of H_2_.

Pure NaBH_4_ samples were analyzed with three different calorimetric methods: TPD, DSC-Netzsch and DSC-Setaram, and TGA (Figure 1). Each of these techniques accessed useful information and presented experimental limitations that might lead to different decomposition behaviors of the samples.

The in-house TPD and the Netzsch DSC (blue and black lines in Figure 1) showed the maximum of the melting point of NaBH_4_ to occur at around 503 ${}^{\circ }$C, in agreement with the literature.

However, the decomposition event happened at higher temperatures with the Netzsch DSC, at about 558 ${}^{\circ }$C, compared to the 534 ${}^{\circ }$C of the TPD curve. The reason for this discrepancy was the fact that the TPD analysis was taking place in a dynamic vacuum, while the Netzsch DSC measured under an Ar flow of 20 mL/min. The Ar flow cooled down the surroundings of the sample, making it more difficult to achieve the necessary temperature to decompose (more heat needed to be applied to decompose the material).

Larger differences were observed between these two techniques and the Setaram DSC analysis. On the one hand, the Setaram DSC technique only showed the NaBH_4_ melting event at 509 ${}^{\circ }$C. The reason for this was that the measurements were generally carried out in closed stainless steel (SS) crucibles. These were high pressure crucibles without a venting hole, and therefore, it was likely that the desorbed gas/H_2_ built pressure inside the crucible and hindered the gas evolution, stopping the decomposition process. On the other hand, the melting point was observed to happen at about 6 ${}^{\circ }$C higher than by the TPD and Netzsch techniques. The reason for this shift was related to both the different experimental environments (vacuum and Ar flows of 15 and 20 mL/min) and the different equilibrium pressure imposed by the closed crucible.

The TGA data showed the expected single-step decomposition corresponding to H_2_ to maximize beyond 600 ${}^{\circ }$C and with its onset at 505 ${}^{\circ }$C.

#### 2.2.2. Temperature-Programmed Desorption Results

**Figure 2:**
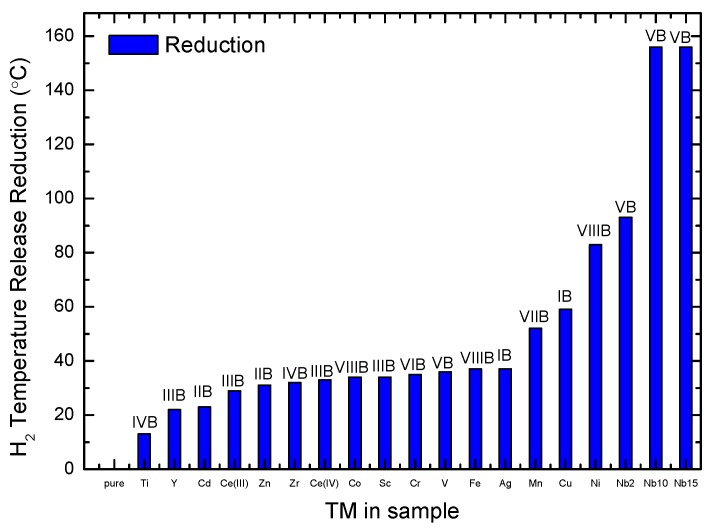
Temperature difference between the main decomposition peak of the samples with additives and that of pure NaBH_4_ as observed by TPD.

Table 2 summarizes the data obtained by TPD for all the samples. The most important peaks were the main decomposition and melting events of NaBH_4_. Furthermore, Figure 2 shows the influence of the TM fluoride additive on the NaBH_4_ decomposition temperature as measured by TPD and represented as the difference in the temperature between the decomposition peak of the sample with additive and that of pure NaBH_4_.

As seen in the figure, prominent reductions in the decomposition temperature corresponded to MnF_3_, CuF_2_, NiF_2_, and NbF_5_, with the largest reduction for 10 and 15 mol% NbF_5_, where the decomposition already occurred at 379 ${}^{\circ }$C. The least influence on the decomposition behavior of NaBH_4_ was observed for TiF_4_ and YF_3_.

However, this destabilization performance could not be correlated to a single fluoride property. On the one hand, MnF_3_, CuF_2_, and NiF_2_ had a relatively high enthalpy of formation, ΔE${}_{form}$, suggesting that less energy was required to mix and react with the borohydride. On the other hand, NbF_5_ had the highest metal oxidation state and the lowest fluoride melting point. The latter property had a strong effect during ball milling as it enhanced the effective surface area for reactions between the fluoride and the borohydride to occur. The high oxidation state of the metal then provided an electronic environment with an abundance of available e${}^{-}$ to assist in further chemical reactions.

By increasing the amount of NbF_5_ additive from 2 to 10 mol%, the decomposition temperature of the remaining NaBH_4_ decreased from 442 to 379 ${}^{\circ }$C. Larger amounts of fluoride additive led to no change in the decomposition features, indicating that there was an optimal amount of additive of 10 to 15 mol% before NaBH_4_ disappears.

The influence of different oxidation states was represented by the CeF_3_ and CeF_4_ cases. For these samples, the TPD data showed that the TM with higher oxidation state resulted in a slightly higher decomposition temperature.

The TPD signals corresponding to diborane species (*m*/*z* = 26, 27) were found to be two orders of magnitude weaker than those for hydrogen (*m*/*z* = 2) for the whole temperature range and for all the investigated samples. No indication of fluoride release was found. Overall, the hydrogen release temperature could not be correlated with the Pauling electronegativity of the TM (χρ) as it was reported for selected TM chlorides on a study on NH_3_BH_3_ [42].

#### 2.2.3. Closed Crucible DSC-Setaram Discussion

**Figure 3:**
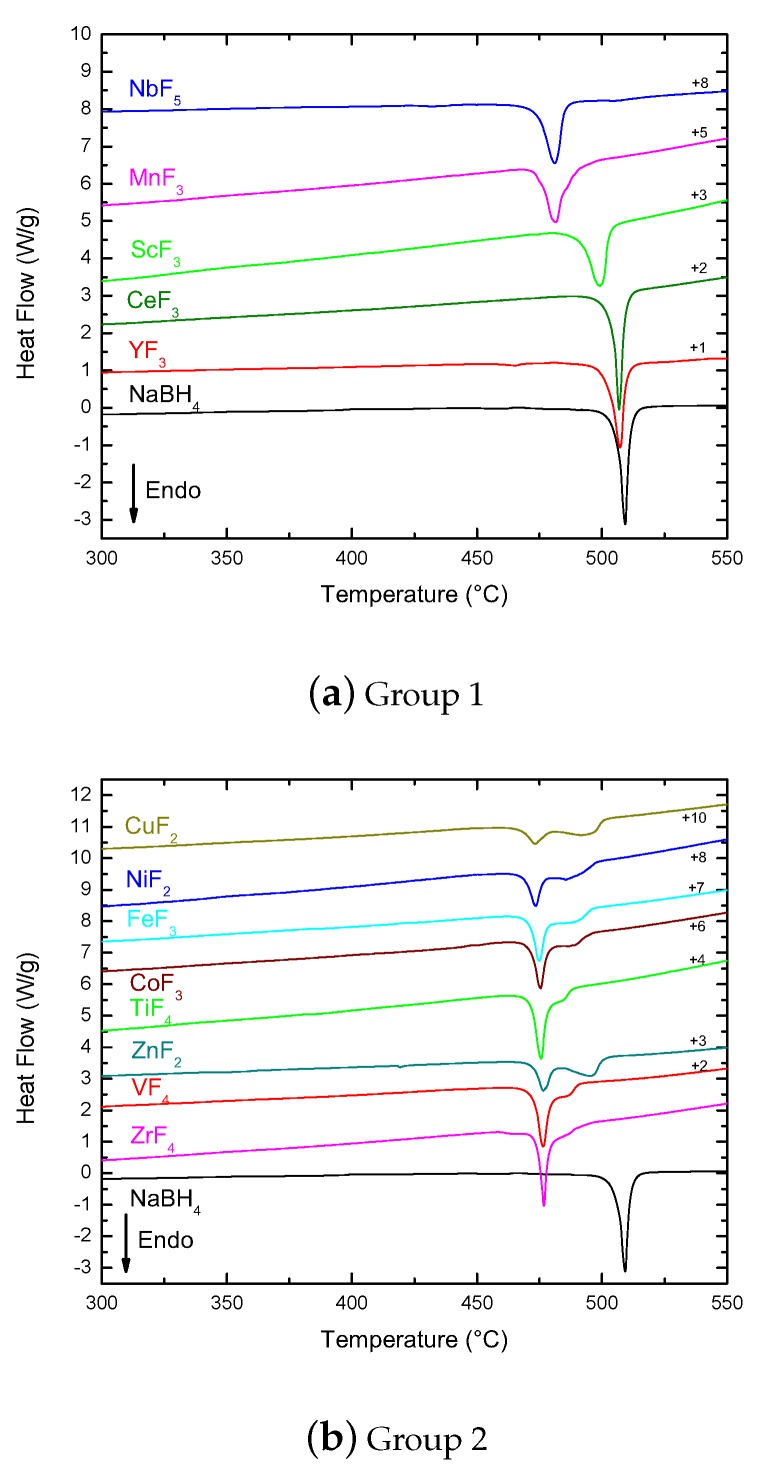

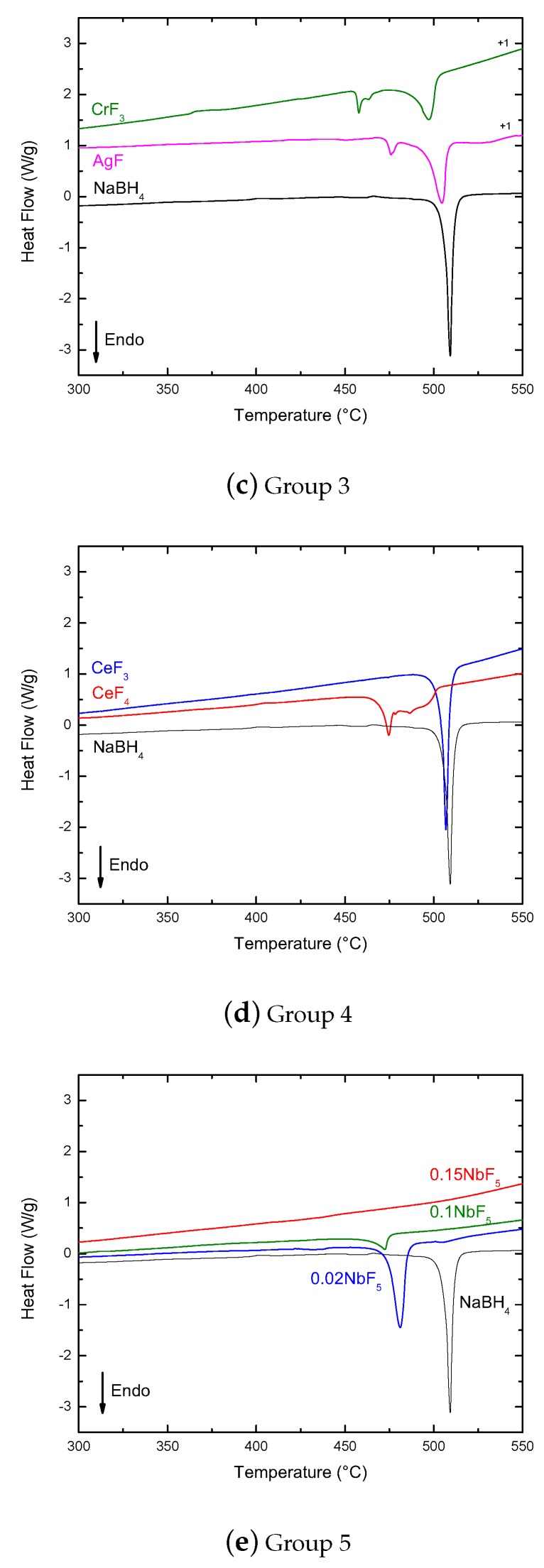
DSC-Setaram corresponding to the samples distributed in different groups based on their behavior. The numbers on the right-hand side indicate the shift applied to the data for plotting.

As discussed in Section 2.2.1, DSC-Setaram data of pure NaBH_4_ showed a strong endothermic event occurring at 509 ${}^{\circ }$C that corresponded with melting. Since the Setaram measurements were done in closed SS crucibles (closed system), the decomposition event at higher temperatures was hindered and not seen. The same effect was expected for the samples containing fluoride additives. In Figure 3, the samples are grouped based on DSC-Setaram measurements.

ScF_3_, YF_3_, and CeF_3_ showed a single endothermic peak attributed to the melting of NaBH_4_, but occurring at slightly lower temperatures (NaBH_4_ 509 ${}^{\circ }$C $>YF_{3}507{\;}^{\circ }$C $\geq CeF_{3}507{\;}^{\circ }$C $>ScF_{3}499{\;}^{\circ }$C).

The ScF_3_ melting feature was broader and asymmetric compared to the narrower peaks of YF_3_ and CeF_3_. From the PXD data in Section 2.1, it was found that the first two samples contained metallic Sc and Y, respectively, but no indications of metallic Ce were observed. Thus, the presence of metallic TM (Sc or Y, respectively) did not explain the different melting profiles. Moreover, the presence of metallic TM did not seem to influence the melting of NaBH_4_ as seen by DSC-Setaram. On the other hand, the asymmetry of some melting peaks could be interpreted as the overlapping of the melting of NaBH_4_ with other intermediate phases formed during heating, as well as by a small gas release, which was weakened in the SS closed system.

The same asymmetry was seen in the DSC-Setaram of MnF_3_ and NbF_5_, which still appeared as single peaks, but at much lower temperatures than the melting point of pure NaBH_4_ (481.5 ${}^{\circ }$C and 481 ${}^{\circ }$C, respectively). These were samples that contained NaBF_4_ (Table 1), which crystallized in a different space group than NaBH_4_ and seemed to have a prominent effect on the melting point. In the case of MnF_3_, shoulders at both sides of the main peak also indicated the overlapping of events related to the presence of different phases and hindered gas release.

The second group of samples (Group 2 in Figure 3) showed a strong decrease of the melting temperature of the ball-milled samples, which now appeared between 473 and 477 ${}^{\circ }$C. In addition, the range of melting temperatures in this group showed a different level of interaction between the TM fluoride additives and NaBH_4_ and their role in disturbing the intermolecular forces in the borohydride. The samples also showed similar second features after the melting peak, between 482 ${}^{\circ }$C at the shoulder in TiF_4_ and the single peak at 496 ${}^{\circ }$C in ZnF_2_. This second feature occurred at too low temperatures to be associated with NaBH_4_ decomposition, which was also hindered by the SS crucibles, avoiding gas release. Therefore, it could only be interpreted as processes occurring on intermediate phases created in the mixture during heating. This was also confirmed by the fact that as the intensity of the melting feature decreased, the stronger the feature at larger temperatures became, indicating that some of the NaBH_4_ in the mixture after milling reacted during heating.

A different behavior was seen in the DSC-Setaram of the remaining samples, AgF and CrF_3_. Their melting points were not strongly changed from that of NaBH_4_, suggesting a small effect by the presence of NaBF_4_ after milling with AgF. On the other hand, features at lower temperatures than their melting peaks indicated the presence of other intermediate phases and processes happening during the low temperature stages of heating.

All in all, the majority of the fluorides studied in this work had an influence on the melting temperature of NaBH_4_. This was particularly true for all the second period TMFs, but also for MnF_3_ and NbF_5_.

The least effective in decreasing the melting temperature, as measured by DSC-Setaram, were YF_3_ and CeF_3_, and the most effective were MnF_3_, NbF_5_, NiF_2_, and CuF_2_. These latter fluorides were the same fluorides that led to the highest hydrogen releases as observed by TPD (Figure 2).

The DSC-Setaram data also showed a significant difference between the CeF_3_ and CeF_4_ samples (Group 4 in Figure 3). While the lower oxidation state compound only decreased the melting temperature of NaBH_4_ slightly, the higher oxidation state showed a feature at 475 ${}^{\circ }$C that could be assigned to the melting and a broad region of overlapping events between 477 and 500 ${}^{\circ }$C. The comparison between the DSC-Setaram behavior of CeF_4_ and the other tetravalent fluorides, VF_4_ and ZrF_4_ (Figure 3, Group 2) showed that CeF_4_ had a smaller effect on the melting point of NaBH_4_. In the case of the trivalent fluorides, MnF_3_, FeF_3_, and CoF_3_ showed the strongest decrease of the melting point (close to 475 ${}^{\circ }$C), while CrF_3_ and ScF_3_ showed a smaller effect and CeF_3_ and YF_3_ the smallest effect.

On the other hand, the increase of NbF_5_ content in the mixture had the effect of reducing the intensity of the melting peak, as well as reducing the melting temperature. For the 15 mol% NbF_5_ sample, the melting peak in the DSC-Setaram disappeared completely, indicating that the amount of NaBH_4_ available was small (Group 5 in Figure 3). This was confirmed by the PXD data, which showed a decrease in the content of NaBH_4_ after ball milling of about 40 % or ca. 59 wt% (Table 1).

#### 2.2.4. DSC-Netzsch Discussion

**Figure 4:**
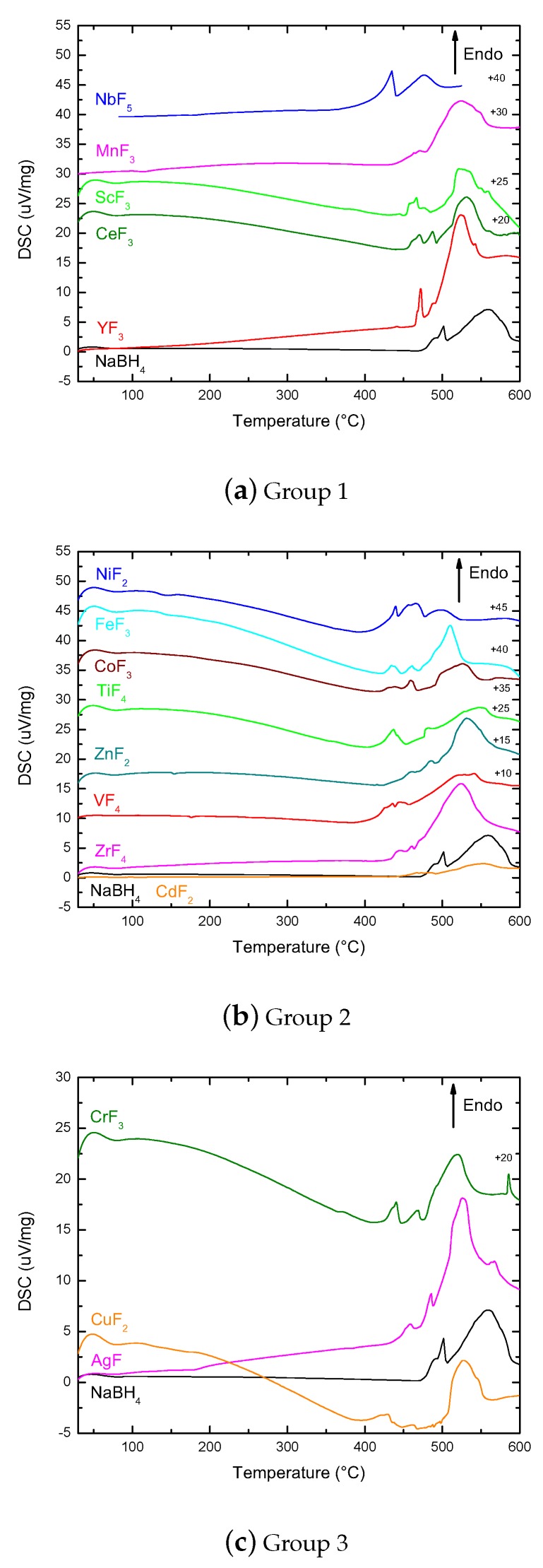

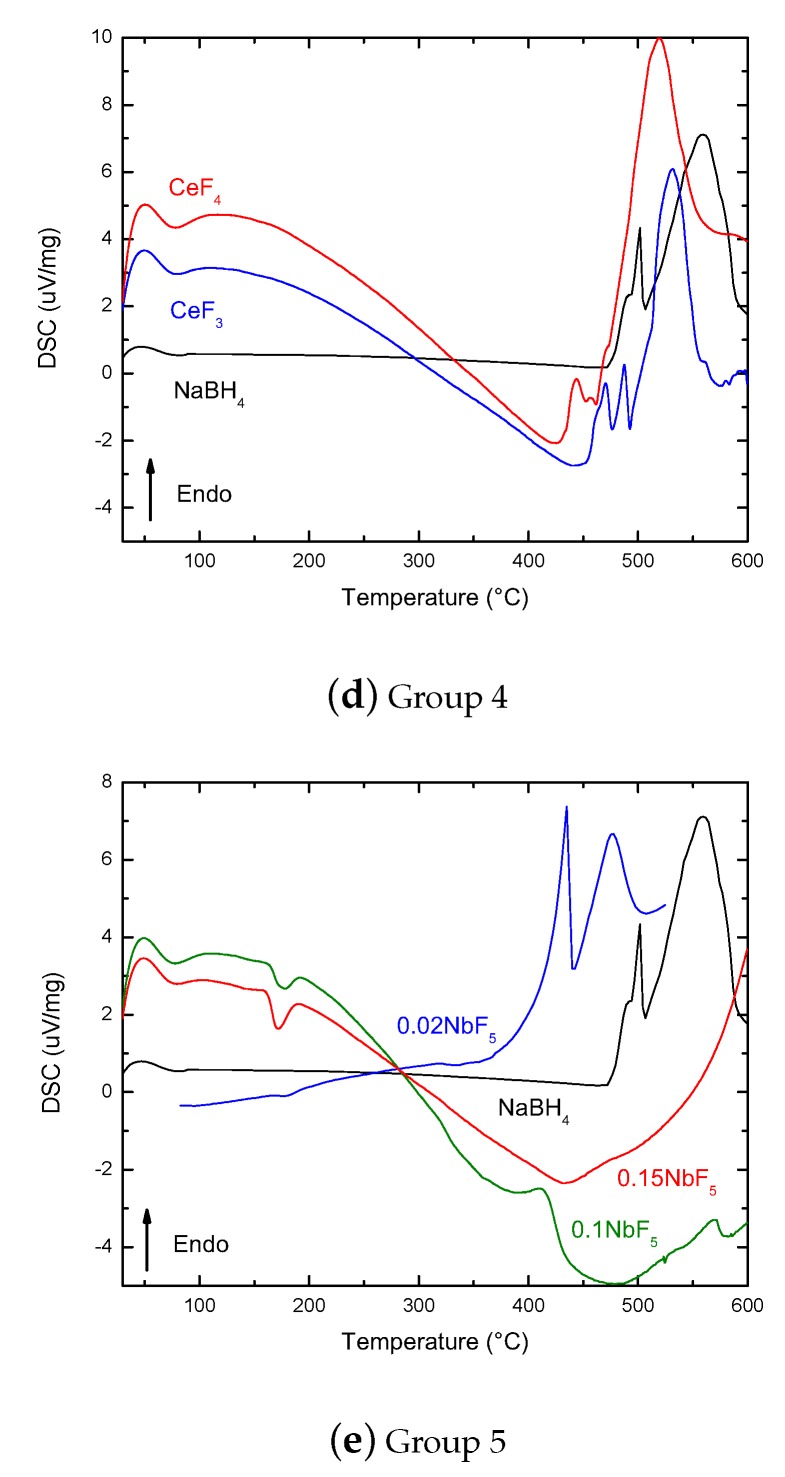
DSC-Netzsch corresponding to the samples distributed in the same different groups as in Figure 3. The numbers on the right-hand side indicate the shift applied to the data for plotting

DSC-Netzsch data complemented the findings by TPD and DSC-Setaram by showing the different calorimetric events during heating in the milled samples as presented in Figure 4.

Both TPD and DSC-Netzsch showed a double feature corresponding to the melting and subsequent decomposition of remaining NaBH_4_ for the samples in the first group. These were both hydrogen release events, with less gas being released during melting in the case of MnF_3_. This was also corroborated by TPD. For ScF_3_ and CeF_3_, the melting regions were made of more than one feature in the DSC-Netzsch data. The extra features were not seen by TPD and therefore corresponded to phase transformations without gas release.

From this group of samples, NbF_5_ was the one with the lowest melting point and the lowest decomposition temperature for the remaining NaBH_4_, in agreement with both TPD and DSC-Setaram results.

The second group of samples showed a heterogeneous behavior in DSC-Nezsch (Group 2 in Figure 4). This was consistent with the results by TPD and DSC-Setaram showing the variety of interactions between the different TM fluorides and NaBH_4_. Like in the previous group, the presence of extra features in the melting area mostly indicated phase transformations without gas release, except for ZnF_2_ and NiF_2_, which showed a TPD shoulder at lower temperatures than the melting of the NaBH_4_ feature. These features corresponded to the growing shoulder observed by DSC-Setaram in Figure 3.

The group made of CrF_3_ and AgF showed the presence of extra DSC features below the melting temperature of NaBH_4_ in both Setaram and Netzsch data. In the case of CrF_3_, the lowest temperature feature corresponded with a gas release shoulder in TPD, while the event in AgF was a phase transformation without gas release. An extra feature at 580 ${}^{\circ }$C for CrF_3_ was only seen by DSC-Netzsch. This also corresponded to a phase transformation without gas release.

The comparison between CeF_3_ and CeF_4_ confirmed the results by DSC-Setaram about the lower oxidation state being less efficient to decrease the melting and decomposition temperatures of NaBH_4_ (Group 4 in Figure 4). Higher oxidation state systems such as CeF_4_ packed more F${}^{-}$ ions around the Ce${}^{+}$ compared to CeF_3_. This caused a decrease of the melting point from 817 to 650 ${}^{\circ }$C that made the TMF more susceptible to reactions with NaBH_4_ during milling. This coincided with the fact that the additive with the highest oxidation state, NbF_5_, resulted in some of the lowest hydrogen melting and desorption temperatures observed. On the other hand, the increase of NbF_5_ content in the mixture led to the decrease of the intensity of the melting and decomposition features. For 15 mol% of NbF_5_, the DSC features were lost (Group 5 in Figure 4).

### 2.3. Thermogravimetric Analysis

**Figure A2:**
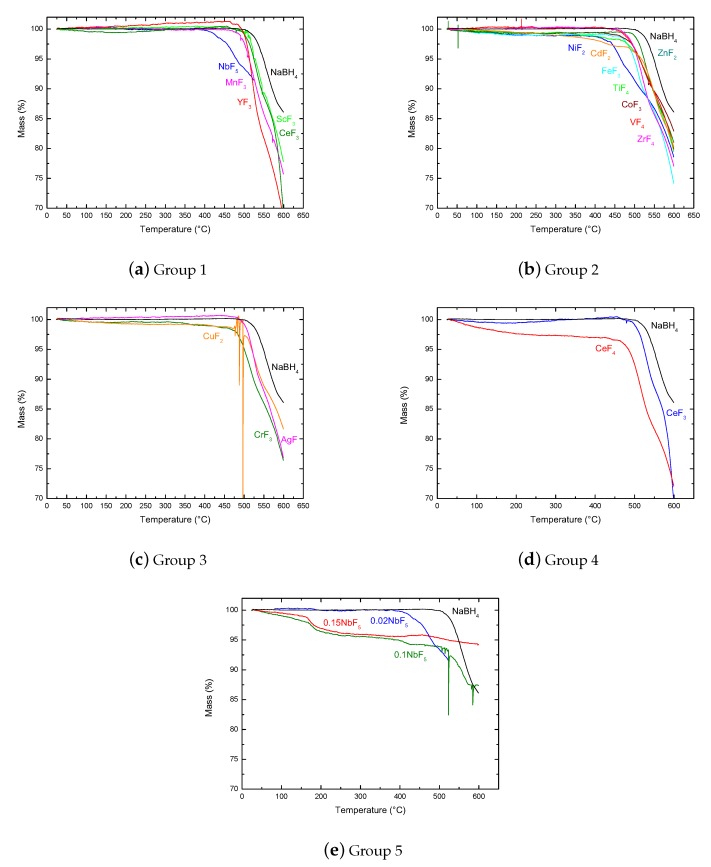
TGA corresponding to the samples distributed in the different groups discussed in the text.

The TGA data showed that for most of the samples, significant mass losses did not start until about 470 ${}^{\circ }$C (see Figure A2 in Appendix A). The most notable exceptions were the NbF_5_ and NiF_2_ samples starting at about 400 ${}^{\circ }$C. Below this temperature, the largest mass loss was seen for NbF_5_, 10 and 15 mol%, with 3.5 and 3.6 wt%, respectively, and for CeF_4_, with 1.4 wt%. All other samples showed mass losses below 1 wt% for the same temperature range. Based on the TPD and DSC results, the mass loss observed below the melting of NaBH_4_ was related to intermediate phases formed during the heating process involving NaBH_4_.

**Figure 5:**
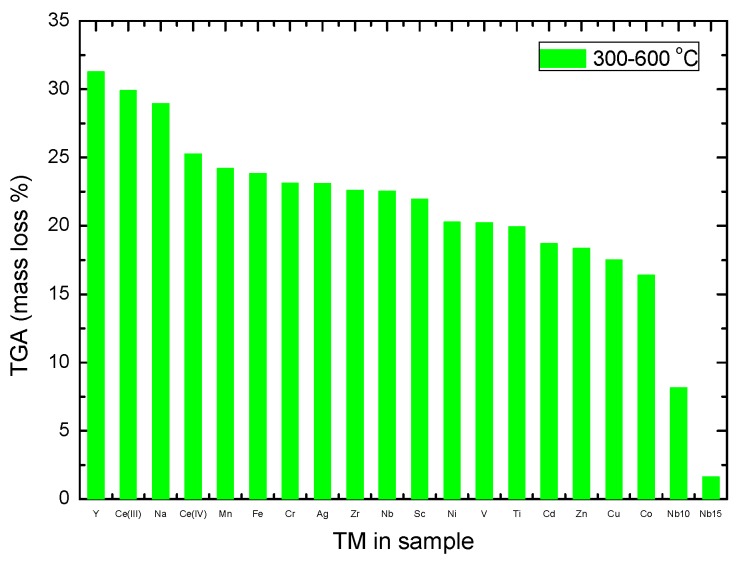
TGA data showing the mass loss between 300 and 600 ${}^{\circ }$C for the samples with additive.

For the samples containing 2 mol% of TM fluoride, the largest mass evolution between 300 and 600 ${}^{\circ }$C was seen for the YF_3_ case (31.3 wt%), while the smallest mass loss was seen for the CoF_3_ sample (16.4 wt%) (Figure 5). These mass losses were larger that the gravimetric capacity of NaBH_4_ (10.6 wt%).

Thus, the mass loss in this temperature range included gas released during melting and decomposition of NaBH_4_, as well as gas release events related to other phases formed by the reaction of the fluoride and the borohydride. This might include a substantial evaporation of Na [43].

The difference between CeF_3_ and CeF_4_ was a decrease of the mass loss for the higher oxidation state: 30 to 25.3 wt% between 300 and 600 ${}^{\circ }$C. When increasing the amount of NbF_5_ from 2 to 15 mol%, the mass loss went from 22.5 to 1.6 wt% in the same temperature range. This indicated that for the higher content of NbF_5_, a larger portion of the hydrogen contained in the mixture with NaBH_4_ was released during the ball milling process due to the low melting point of the fluoride. In order to increase the hydrogen yield, the amount of NbF_5_ had to be lower than 2 mol%, which would also affect the melting and decomposition temperatures.

The TGA data showed that even if NbF_5_ was one of the most efficient additives to decrease the melting and decomposition temperatures of NaBH_4_, as seen by TPD and DSC, its usefulness for hydrogen storage was hindered by its reactive behavior and the small yield of hydrogen obtained from the milled mixture. The results also showed that a TM fluoride such as MnF_3_ produced a desirable destabilization of NaBH_4_, while still giving high hydrogen yields of 24.2 wt% between 300 and 600 ${}^{\circ }$C (Table 3).

## 3. Materials and Methods

Mixtures containing pure NaBH4 (Sigma Aldrich, 99%) and a commercially available anhydrous transition metal fluoride (TMF, Sigma Aldrich: in 1:0.02 molar ratios (2 mol%)) were ball milled in Ar atmosphere using a Fritzch Pulverisette 7 Planetary Mill (300 rpm) and hardened stainless steel vials and balls (Table 4). The samples included the complete first period TM and the available YF_3_, ZrF_4_, NbF_5_, AgF, and CdF_2_ from the second period, as well as CeF_3_ and CeF_4_. The lanthanide metal Ce was chosen due to its light weight. In the NbF_5_ case, additional molar ratios of 1:0.10 and 1:0.15 (10 and 15 mol%) were also prepared to study the destabilization effect of increasing the amount of additive. The fluoride name was used throughout the text to identify the NaBH_4_ + TMF mixture.

All the samples were treated equally and were milled for 1 h with a ball-to-powder ratio of 40:1. Both hardened stainless steel vials and balls (10 mm ϕ) were used for the milling. Sample handling was carried out in MBraun Unilab glove boxes filled with purified argon (<1 ppm O_2_, H_2_O) to avoid contamination.

Powder X-ray diffraction (PXD) patterns were collected in transmission mode using CuKα radiation (λ = 1.5418 Å) in a Bruker AXS D8 Advance Diffractometer equipped with a Göbel mirror and a LynxEye${}^{TM}$ 1D strip detector. The samples were packed in sealed boron glass capillaries (0.5 and 0.8 mm ϕ) in Ar atmosphere. These were kept rotating during measurements to decrease preferred directionality effects. Small amounts of pure Si were added to some samples as internal standard (ABCR, APS 1-5 micron, 99.999%) to determine the instrumental off-set. Acquisition of data were restricted to the 2θ = 5–80${}^{\circ }$ range, with Δ2θ = 0.02${}^{\circ }$ and 2 s/step scanning rates.

Differential scanning calorimetry (DSC) measurements were performed both in a Setaram Sensys DSC and a Netzsch STA 449 F3 Jupiter instrument that also performed simultaneous Thermogravimetric Analysis (TGA). In the Setaram case, 50 mg of sample were put into high pressure stainless steel crucibles that were heated up to 600 ${}^{\circ }$C with an Ar flow of 15 ml/min and a heating rate of 2 ${}^{\circ }$C/min. For the simultaneous TGA and DSC experiments performed in the Netzsch instrument, 3 to 5 mg samples were placed in Al crucibles with pierced lids and heated between 30 and 600 ${}^{\circ }$C, with a heating rate of 2 ${}^{\circ }$C/min under argon gas flow (100 mL/min).

The different experimental conditions of the DSC experiments were chosen to provide as much complementary information as possible on the effects induced by the TM fluorides on the NaBH_4_.

Additional temperature-programmed desorption (TPD) with residual gas analysis (RGA) data were collected from approximately 25 mg of sample with an in-house built setup under vacuum conditions (10${}^{-5}$ mbar). Heating ramps between RT and 600 ${}^{\circ }$C at a constant heating rate of 2 ${}^{\circ }$C/min were used. RGA data were obtained with a MULTIVISON IP detector system coupled to a PROCESS Eye analysis package from MKS Instruments.

## 4. Conclusions

Transition metal fluorides from the first and second periods of the periodic table milled with NaBH_4_ in a 0.02:1 molar ratio exhibited a destabilizing effect that led to the decrease of the melting and the decomposition temperatures of the borohydride below 505 ${}^{\circ }$C and 535 ${}^{\circ }$C, respectively.

- In particular, NbF_5_ and MnF_3_ were very good destabilizers of NaBH_4_, with a 30 ${}^{\circ }$C decrease of its melting temperature and a 50 to 57 ${}^{\circ }$C decrease of its decomposition temperature, while still giving high decomposition gas yields in the 300 and 600 ${}^{\circ }$C region of 24.2 and 22.5 wt%, for 2 mol% of MnF_3_ and NbF_5_, respectively, that might include evaporation of Na.
- In addition, the strong reactivity of NbF_5_ meant that the yield of hydrogen from a mixture with NaBH_4_ decreased strongly with increasing fluoride amount (1.6 wt%, for 15 mol% of NbF_5_), since most of the hydrogen was lost during the ball milling process.
- Increasing the additive amount from 2 to 10 and 15 mol% led to the loss of the NaBH_4_ and therefore the loss of hydrogen yield during thermal decomposition.
- Higher oxidation states of the metal in the fluoride were more efficient in reducing the melting and decomposition temperatures of NaBH_4_. This was confirmed by the comparison between CeF_3_ and CeF_4_ (506 and 502 ${}^{\circ }$C, respectively), but also by the results showing NbF_5_, the TM fluoride with highest oxidation state, being one of the most efficient destabilizers.
- An increase of the oxidation state also seemed to lead to a decrease of the gas yield in the 300 and 600 ${}^{\circ }$C region, with 29.9 and 25.3 wt%, for CeF_3_ and CeF_4_, respectively).

It was found that the destabilizing performance of the studied fluorides depended on a combination of their properties rather than on a single parameter. Higher fluoride melting points required higher energy ball milling conditions than lower melting points to achieve similar chemical interactions with NaBH_4_ during ball milling, while smaller enthalpies of formation and higher metal oxidation values enhanced the chemical interaction further during and after the ball milling process.

Future studies are envisioned to understand how the different properties act on the most successful fluorides found in this work.

## Figures and Tables

**Table 1:** Composition of the samples before and after ball milling. The first 5 columns show the composition of the ball-milled samples as evaluated by EVA. The two last columns are the calculated wt% of the original physical mixture before ball milling.

Sample	NaBH_4_ (wt%)	TMF (wt%)	NaBF_4_ (wt%)	TM (wt%)	Other (wt%)	NaBH_4_ (wt%)	TMF (wt%)
ScF_3_	95.5	4.5				94.9	5.1
TiF_4_	82.0		18.0			93.9	6.2
VF_4_	77.9	3.6	18.5			93.7	6.3
CrF_3_	95.7	4.0		0.4		94.6	5.5
MnF_3_	55.3		44.7			94.4	5.6
FeF_3_	94.9	5.1				94.4	5.6
CoF_3_	95.8	3.2			1.0	94.2	5.8
NiF_2_	94.5	5.5				95.1	4.9
CuF_2_	88.8	9.8		1.4		94.9	5.1
ZnF_2_	95.1	4.9				94.8	5.2
YF_3_	92.5	7.5				92.8	7.2
ZrF_4_	100					91.9	8.1
NbF_5_ (2 mol%)	88.1		4.6		2.8	91.0	9.0
NbF_5_ (10 mol%)	70.2		8.0		2.4	66.8	33.2
NbF_5_ (15 mol%)	59.0		10.6		2.6	57.3	42.7
AgF	93.7	0.8	2.9	1.0	1.5	93.7	6.3
CdF_2_	90.5	6.6	2.9			92.6	7.4
CeF_3_	97.6		2.4			90.6	9.4
CeF_4_	100					89.8	10.3

**Table 2:** TPD temperatures for the main decomposition and melting events of all the samples together with their temperature difference when comparing to NaBH_4_. The tabulated values correspond to the maxima of the measured data in every peak region. The samples are ordered from top to bottom following Figure 2.

Sample	Decomposition	Difference	Melting	Difference
	${}^{\circ }$C	${}^{\circ }$C	${}^{\circ }$C	${}^{\circ }$C
NaBH_4_	535	0	503	0
TiF_4_	522	13	452	51
YF_3_	513	22	462	41
CdF_2_	512	23	461	42
CeF_3_	506	29	487	16
ZnF_2_	504	31	460	43
ZrF_4_	503	32	460	43
CeF_4_	502	33	457	46
CoF_3_	501	34	452	51
ScF_3_	501	34	455	48
CrF_3_	500	35	464	39
VF_4_	499	36	452	51
FeF_3_	498	37	460	43
AgF	498	37	466	37
MnF_3_	483	52	453	50
CuF_2_	476	59	425	78
NiF_2_	452	83	446	57
NbF_5_ (2 mol%)	442	93	190	313
NbF_5_ (10 mol%)	379	156	315	188
NbF_5_ (15 mol%)	379	156	315	188

**Table 3:** Mass loss measured by TG in the 100–300 ${}^{\circ }$C and 300–600 ${}^{\circ }$C temperature ranges. The tabulated values correspond to the minima of the measured data in every region. Samples are ordered from larger to smaller losses following Figure 5.

Sample	TG Mass Loss %	TG Mass Loss %
	100–300 ${}^{\circ }$C	300–600 ${}^{\circ }$C
NaBH_4_		14.0
YF_3_	0.7	31.3
CeF_3_	0.3	29.9
CeF_4_	1.4	25.3
MnF_3_	0.2	24.2
FeF_3_	0.4	24.0
CrF_3_	0.1	23.1
AgF	0.3	23.1
ZrF_4_	0.4	22.6
NbF_5_ (2 mol%)	0.3	22.5
ScF_3_	0.3	22.0
NiF_2_	0.6	20.3
VF_4_	0.1	20.2
TiF_4_	0.3	20.0
CdF_2_	0.9	19.0
ZnF_2_	0.2	18.3
CuF_2_	0.5	17.5
CoF_3_	0.4	16.4
NbF_5_ (10 mol%)	3.5	8.2
NbF_5_ (15 mol%)	3.6	1.6

**Table 4:** Relevant transition metal fluorides data. Enthalpy values (for the solid phase) from the Peep Database [44].

1st period	ScF_3_	TiF_4_	VF_4_	CrF_3_	MnF_3_	FeF_3_	CoF_3_	NiF_2_	CuF_2_	ZnF_2_
electronic	Ar	Ar	Ar3d${}^{3}$	Ar3d${}^{1}$	Ar3d${}^{3}$	Ar3d${}^{6}$	Ar3d${}^{5}$	Ar3d${}^{8}$	Ar3d${}^{9}$	Ar3d${}^{10}$
melting point/${}^{\circ }$C	1552	377	325	1100	600	1200	927	1474	836	1500
enthalpy of decomposition	−385.2	−394.2	−335.4	−277.2	−256.0	−236.7	−188.9	−157.1	−128.8	−182.7
H/kcal mol${}^{-1}$										
2nd period	YF_3_	ZrF_4_	NbF_5_						AgF	CdF_2_
electronic	Kr	Kr	Kr						Kr4d${}^{9}$5s${}^{1}$	Kr4d${}^{10}$
melting point/${}^{\circ }$C	1387	910	90						435	1110
enthalpy of decomposition	−410.7	−456.8	−433.5						−48.5	−167.4
H/kcal mol${}^{-1}$										
other	CeF_3_	CeF_4_								
electronic	Xe4p${}^{1}$	Xe								
melting point/${}^{\circ }$C	817	650								
enthalpy of decomposition	−403.7	−442.0								
H/kcal mol${}^{-1}$										

## Abbreviations

The following abbreviations are used in this manuscript:

MDPI	Multidisciplinary Digital Publishing Institute
DOAJ	Directory of open access journals
PEMFCs	proton exchange membrane fuel cells
DBFCs	direct boron hydride fuel cells
TM	transition metals
PXD	powder X-ray diffraction
DSC	differential scanning calorimetry
TPD	temperature-programmed desorption
TMF	transition metal fluorides
TGA	thermogravimetric analysis
RT	room temperature
RGA	residual gas analyzer
